# Primo-Vascular System as Presented by Bong Han Kim

**DOI:** 10.1155/2015/361974

**Published:** 2015-08-25

**Authors:** Vitaly Vodyanoy, Oleg Pustovyy, Ludmila Globa, Iryna Sorokulova

**Affiliations:** ^1^Department of Anatomy, Physiology and Pharmacology, College of Veterinary Medicine, Auburn, AL 36849, USA; ^2^School of Kinesiology, Auburn University, Auburn, AL 36849, USA; ^3^Edward Via College of Osteopathic Medicine, Auburn, AL 36849, USA

## Abstract

In the 1960s Bong Han Kim discovered and characterized a new vascular system. He was able to differentiate it clearly from vascular blood and lymph systems by the use of a variety of methods, which were available to him in the mid-20th century. He gave detailed characterization of the system and created comprehensive diagrams and photographs in his publications. He demonstrated that this system is composed of nodes and vessels, and it was responsible for tissue regeneration. However, he did not disclose in detail his methods. Consequently, his results are relatively obscure from the vantage point of contemporary scientists. The stains that Kim used had been perfected and had been in use for more than 100 years. Therefore, the names of the stains were directed to the explicit protocols for the usage with the particular cells or molecules. Traditionally, it was not normally necessary to describe the method used unless it is significantly deviated from the original method. In this present work, we have been able to disclose staining methods used by Kim.

## 1. Introduction

In the early 1960s, Bong Han Kim discovered and described a new vast anatomical vascular system that he believed underpins the acupuncture meridian system. During the three years from 1962 to 1965 Kim published five reports [1–5]. Four of Kim's reports were translated into English as two books [6, 7]. He analyzed and described this new anatomical system that he named as Bonghan system. It was obvious from his publications that Kim had far-reaching scientific goals, but, in 1965, his research ceased, and his fate became unknown [8, 9].

Following Dr. Kim's disappearance, his findings remained dormant for many years. In 2002, the scientific group of Dr. Kwang-Sup Soh initiated a series of experiments that validated many of Kim's results. This research has ignited a new interest in this anatomical vascular system that now is termed primo-vascular system (PVS) [10]. Soh and his colleagues have described the major achievements of Bong Han Kim in the understanding of structure and functions of PVS, as well as the experimental and theoretical results obtained by Kim during the relatively short time [10]. Kim offered a comprehensive picture of primo-vascular system. Kim presented the structural architecture that he had observed, which included organ, tissue, cells, and molecules involved in the function of PVS [7]. A short list of his techniques consisted of microsurgery, light and electron microscopies, and time-lapsed photography. Instead of dye-labeled antibodies he used fluorescent and nonfluorescent histochemistry with a large variety of stains, radioactive tracers, microautoradiography, and emission spectral analysis. He also perfected cell analysis, nucleic acid analysis, ultracentrifugation, cell culture, cell development, tissue regeneration, chromatography, electrophoresis, embryology, physiology, and electrophysiology [6, 7]. Kim gave a vivid description of primo-nodes and primo-vessel, which are two major components of primo-vascular system.

In this work, we have recreated structural models of the PVS node and vessels as they were described by Kim and compare these models with our optical experimental data and results found so far in the literature.

## 2. Terminology

Shortly before the first International Symposium on Primo-Vascular System, which was held in Jecheon, Korea, during September 17-18, 2010, Dr. Kwang-Sup Soh suggested that it would be important to agree upon a single terminology for the Bonghan system. It was agreed that the following terms would be adopted: Bonghan system (BHS) = primo-vascular system (PVS), Bonghan duct (BHD) = primo-vessel (PV), Bonghan corpuscle (BHC) = primo-node (PN), Bonghan ductule = P-subvessel, Bonghan liquor = primo-fluid (P-fluid), and Sanal = p-microcell.

The present work includes detailed presentations of PVS structures that require additional new terms as follows:

 periductium= p-vessel external jacket;
 wall of the Bonghan ductule + outer membrane of Bonghan ductule = external envelope of p-subvessel;
 outer membrane of Bonghan corpuscle = primo-node capsule;
 sanalosome= p-microcell nucleosome;
 sanaloplasm= p-microcell nucleoplasm;
 small nucleus-like structures = small (immature) p-microcell = progenitors of multipotent stem cells;
 large nucleus-like structure = large (mature) p-microcell = multipotent stem cell; sanalization = conversion of cell into p-microcell.

## 3. A Brief Old History

For many, the acupuncture meridian system is nothing more than a network of lines drawn on a body map and labeled with hieroglyphs. For others, the exact positions and locations of acupuncture points and meridians result from a few thousand years' empirical practices of acupuncturists. It seems that no theoretical or anatomical background for the location and morphology of these points is available. On the contrary, there is strong evidence that information on the acupuncture points and meridians is based on the anatomical knowledge used in ancient Chinese surgical practices [11–13]. Anatomical dissections are mentioned in the Huang-Di Nei-Jing Ling-Shu, one of the oldest traditional books relating to Chinese medicine that describes anatomical structures of acupuncture points [14]. The most important source of information and exact anatomical description of acupuncture points is the classic Tong Ren, the Copper Man by Wang Wei-Yi published around 1027 A.D. (cited by Schnorrenberger [11]).

Due to the considerable difference between ancient and modern anatomical nomenclature, it is difficult to comprehend the full extent of the morphological properties of acupuncture points described by ancient scholars. It seems that detailed descriptions of anatomical structures given in the ancient Chinese sources would require microscopic histological examination. It is not known if ancient Chinese medical doctors used any magnifying tools in their anatomical work. The first recorded use of a microscope can be traced 4,000 years back to the Chow-Foo Dynasty. Ancient Chinese text refers to the construction of a magnifying tube filled with water with a refractive single lens in the lower end of this tube. By filling the tube with water at different level, they attained different level of magnification [15]. The highest magnification possible, 150x, would be powerful enough to see many morphological features that can be observed in modern microsurgery. Therefore, it would be very exciting to find a correspondence between ancient and modern anatomical nomenclature to comprehend the vast morphological knowledge that might have been available 2000 years ago. Additionally, there is evidence that acupuncture was practiced in Central Europe over 5200 years ago [16, 17].

Based upon the ancient literature, it is not clear whether ancient Chinese doctors knew the specific morphology of an acupuncture point. However, it is clear that the ancient knowledge of acupuncture meridians was more detailed and explicit. In contrast to the western view of meridians as mere lines on the skin, the classical Chinese text indicates that the meridians, in fact, possess a three-dimensional topology. They run deep inside the human body connecting with internal organs. In ancient Chinese text they are referred to as “Jing Mai” (pulsing vessels) [13] that carry Qi, nutrition, defensive factors, and liquid [18, 19]. The Qi is known to have many different meanings. In most cases, it is interpreted as a special substance or liquid that is in “a constant state of flux and varying states of aggregation” [18]. “Jing” is often translated as “essence.” Congenital Jing is a substance that is received from one's parents at conception and which governs the growth and regeneration processes from the conception to death.

Huang-Di determined that Jing Mai also participated at the earliest stages of the human embryo development. He explains the creation of a human being by the combination of the female ovum Jing with a male sperm Jing. It involves Jing Mai (pulsing vessels) at the unfolding of brain and spinal cord tissues that correspond to the ectoderm layer, one of the three germ layers recognized in embryology [13, 14, 20].

Rephrasing the ancient Chinese concept of acupuncture meridians (Jing Mai), one can say that ancient Chinese medical doctors viewed the meridians as a three-dimensional vascular system. The system is carrying a special liquid (Qi) which contains genetic material (Jing) that is initially obtained after conception.

Ironically, putative primo-vessels have been discovered in Europe before the discovery of lymphatic vessels/system. In 1622, Gasparo Aselli, a professor of anatomy and surgery, Pavia, Italy, found vessels in the mesentery of dog that were filled with a white liquid. These white vessel structures ran through the mesentery and along the surface of the intestines and emitted a milky fluid when cut. Because of the milky appearance of the vessels, Aselli named these structures as* lacteis venis or* milky veins. Aselli stated that the liquid in these vessels is transformed in blood. Later, this statement was rejected by the researchers working with lymphatic vessels [21, 22], but Aselli is still credited as the discoverer of the lymphatic system. It is interesting to note that Aselli may have, in fact, described the existence of primo-vessels because the hematopoiesis that he described was also documented to be in the internal primo-node. It was later demonstrated by Kim [5, 7].

In 1874, Louis-Antoine Ranvier, who discovered nodes of Ranvier, also described unusual structures in the omentum of new born animals. He described these structures as being elongated or round, occasionally branched elements, containing red and white corpuscles [23]. About a quarter of the century later, Marchand [24] also found and described similar structures in the animal omentum. He described them as elongated elements, accompanied by blood vessels and associated with the production of all types of blood cells. In 1909, analyzing the interaction of various vital stains and colloidal metals with these structures, Goldman [25] reported that this type of “reticular-endothelial” system can be identified by trypan blue stain. Trypan blue belongs to the group of benzidine dyes containing trypan red and pyrrhol blue. He indicated that the pyrrhol blue particles create granular inclusion in the cells of these systems, which he named as “pyrrhol cells.”

Maximow, the scientist who coined the name “stem cell” [26], also described structures and functions of hematopoietic systems as scaffolds or niches for stem cell maturation and subsequent development of blood cells: “*… they appear as sharply outlined, polymorphous, flat, spindle shaped or branched elements, often containing inclusions and are then easily distinguishable from the fibroblasts. They may also assume a flat shape and line blood or lymph channels. More than by a peculiar histological structure all these elements are characterized by a series of very important functional properties. Being endowed with ample prospective potencies they can produce, provided external conditions are favorable for hemocytoblasts* (hematopoietic stem cells)* and different types of blood cells. It is difficult to choose a suitable general name for these elements of the connective tissue, remaining throughout the whole life in an undifferentiated embryonic condition. They represent a vast cell system, distributed all over the body, over various organs and assuming, according to their position, manifold histological aspects. As we have seen, single parts of this cell system have been described by various investigators under different names. However, the idea of the close interrelation or even identity of all these cells, the fact that they form one entity, one vast group of elements with a very prominent function in the body, has made headway slowly*” [27]. In this relatively short paragraph, Maximow described the essence of the primo-vascular system that was independently discovered by Bong Han Kim, which he subsequently named as Bonghan system [1–7, 28, 29].

## 4. Summary of Kim's Findings on Primo-Vascular System

In the 1960s, Bong Han Kim, a North Korean scientist and professor of Pyongyang Medical College, suggested that the superficial acupuncture meridian system represented a fundamental vascular system. He injected radioactive phosphorus (P^32^) into a rabbit primo-node and documented that the P^32^ tracked or followed the acupuncture meridians [6]. Kim revealed that he separated DNA granules (p-microcells) from the primo-vessels and stimulated their proliferation under artificial conditions [7].

Importantly, radioactive visualization of the acupuncture meridians was reported again [30–32]. The research teams injected the radioisotope technetium (Tc^99^) at acupoints and described that the effective radiotracer pathways coincided with acupuncture meridians. A physical reality of acupuncture meridians was also confirmed by the increase in electroconductivity, hydraulic conductance, and propagation of acoustic waves [32–34]. Furthermore, infrared light delivered at acupoints was shown to travel in tracks detectible on the skin and these tracks correspond to traditional acupuncture meridians [35].

Three uniquely critical anatomical structures were reported as having both distinctive functions and structures. These are (1) superficial nodes positioned at the acupuncture points; (2) profound nodes in deep tissues located in and around blood and lymphatic vessels and internal organs; and (3) primo-vessels that connect all nodes which comprises the primo-vascular system. Kim proposed that p-microcells, DNA-containing granules, mature in the Bonghan system and produce small cells that are transported through ducts to replace aged and dying cells. These small cells, as described by Kim, behaved like multipotent stem cells [7].

## 5. Primo-Vascular Node and Vessels 

According to Kim, a primo-vessel connects primo-nodes, and a primo-node is linked with primo-vessels.

### 5.1. Primo-Vessels

Kim recognized four different types of primo-vessels as follows. (1) The primo-vessels floating in the blood and lymphatic vessels were named as the internal (*intravascular*) primo-vessels. (2) The primo-vessels distributed on the surface of the organs, independent of the blood and lymphatic vessels and neuronal axons, were named as the* intraexternal* primo-vessels. (3) The primo-vessels running along the outer surface of the walls of blood and lymphatic vessels were named as the external (*extravascular*) primo-vessels. The external primo-vessels sometimes run, either independently of blood vessels or along neuronal axons. (4) The primo-vessels distributed in the internal and the peripheral nervous system, running inside the central canal of the spinal cord and the cerebral ventricles, were named as the* neural* primo-vessels [7].

**Figure 1:**
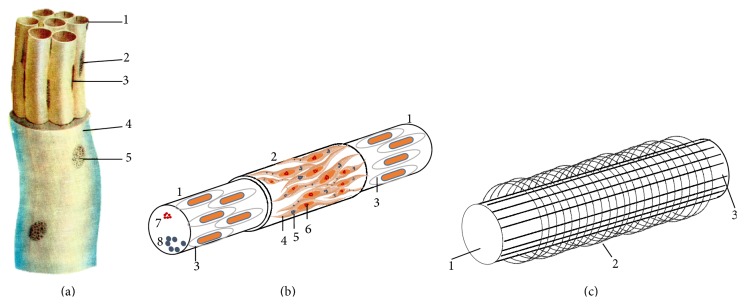
Illustration of the primo-vessel and p-subvessel. (a) Primo-vessel. 1: primo-subvessel; 2: cell nucleus of the outer membrane; 3: nucleus of endothelial cell; 4: external jacket of primo-vessel; 5: nucleus of jacket endothelial cell [7]. (b) Diagram of primo-subvessel. 1: wall of subvessel formed by endothelial cells; 2: outer membrane of subvessel; 3: endothelial cell with rod-shaped nucleus; 4: spindle-shaped cell with ellipsoidal nucleus; 5: fine basophil granules in the cytoplasm; 6: fine chromatin granules inside nucleus; 7: basophil granules inside the subvessel; 8: p-microcells. (c) Diagram of subvessel fibers. 1: primo-subvessel; 2: fine transversal fiber; 3: longitudinal fiber.

The anatomical structure of different types of primo-vessels varies, but all of them share some common features. The primo-vessel is composed of 1–20 p-subvessels of 3–25 *μ*m in diameter (Figure 1(a)) [7]. The bundle of p-subvessel of the primo-vessel is laid into an external jacket composed of endothelial cells with 6–12 *μ*m round or oval nuclei.

The intraexternal primo-vessels, the simplest for microscopic observations, run openly on the surface of the internal organs in the thoracic and abdominal cavities and create a network, giving branches to various internal organs. Analyzed under a microscope, the nonfixed sample of intraexternal primo-vessel is revealed as a translucent, milk-white structure, which is covered with a very thin, transparent membrane [7].

### 5.2. P-Subvessels

**Figure 2:**
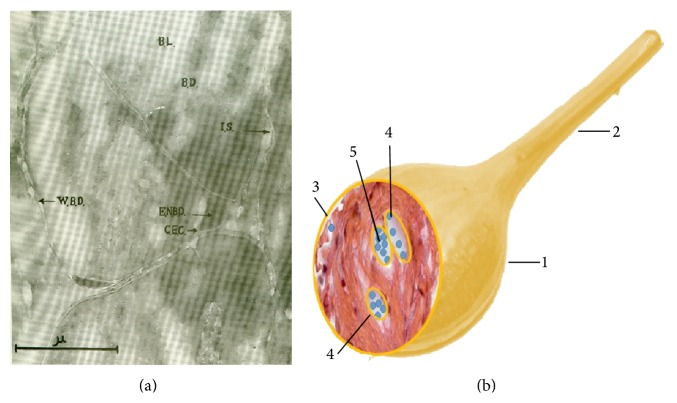
Primo-vessel and node. (a) Electron micrograph of the internal primo-vessel (cross section) (×42,000). BL: primo-fluid, BD: p-subvessel, IS: interstitial substance, WBD: external envelope of p-subvessel, ENBD: endothelial nucleus of the p-subvessel, and CEC: cytoplasm of endothelial cell [7]. (b) Diagram of the transversal section of a primo-node. 1: Primo-node; 2: primo-vessel; 3: node capsule; 4: lumens; 5: p-microcells.

The external envelope of the p-subvessel is composed of two layers (Figure 1(b)): the wall of endothelial cells with a rod-shaped nucleus of 15–20 *μ*m and the outer membrane containing spindle-shaped cells with ellipsoidal nucleus of 13–27 *μ*m long and 4-5 *μ*m thick that are similar to smooth muscle cells. These cells are characterized by fine basophil granules in the cytoplasm and fine chromatin granules inside the nuclei. The thickness of the internal wall, when observed by an electron microscope, is only 0.1–0.2 *μ*. The p-subvessels are surrounded by fine, longitudinal, and circular fibers crossing each other (Figure 1(c)). A high magnification of a primo-vessel was imaged by Kim using transmission electron microscopy [7] and presented in Figure 2(a). It clearly shows that the external envelope of p-subvessel is a two-layer structure. The micrograph also shows that the endothelial nucleus (ENBD) of the p-subvessel belongs to the internal layer. So it is safe to speculate that the external layer is the outer membrane of p-subvessel that contains muscle-like cells.

### 5.3. Primo-Nodes

Kim classified the primo-nodes into two categories: the superficial and the profound primo-nodes. The profound primo-nodes again were further categorized into the internal (intravascular), external (extravascular), intraexternal, neural, and intraorganic primo-nodes. The primo-vessel departs the intraorganic primo-node branches and extends to several small intraorganic nodes (called the terminal primo-nodes). The fine p-subvessels running out of the terminal primo-nodes (terminal p-subvessels) are directly linked to the cell nuclei. The internal primo-node is linked by internal primo-vessels, the external node by external vessels, and the superficial node by superficial vessels. The intraexternal and the neural nodes are also linked by the vessels of relevant names, respectively [7].

The primo-nodes have various shapes (round, oval, or multifaceted). One to a few p-vessels enter and exit the node. The most typical primo-nodes have oval shape of 0.1–0.5 mm over the short and 0.5–1 mm over the long axis. Both node's ends are linked to primo-vessels of 3–6 cm long and 40–100 *μ*m in diameter (Figure 2(b)). The primo-node is the anastomosis of widened and branched p-subvessels covered with a 5–40 *μ*m thick capsule. A p-vessel bundle of the incoming (afferent) vessel enters into the node, branches into additional bundles, and fills the node interior with tightly twisted and bent bundles. P-subvessels converge, narrow, and come out from the node as a single efferent primo-vessel. An enlarged p-subvessel which is called the sinus of the node harbors basophil granules and p-microcells.

Reticular fibers composed of collagen secreted by reticular cells are part of the primo-node. Reticular fibers intersect to form a fine network that functions as a supporting lattice in the primo-nodes and vessels [7].

### 5.4. Primo-Fluid inside PVS

Kim found that p-fluid contains large concentration of nucleic acids, 3.12–3.40% of total nitrogen, 10–0.17% of nonprotein nitrogen, 0.57–1.00% of lipids, 0.10–0.12% of reduced sugars, 170.4 mg% of total hyaluronic acid, 0.22–0.4 *μ*g/g of epinephrine (adrenaline), and 0.6–1.5 *μ*g/g. It also contains norepinephrine (noradrenaline) and gonadal hormone, estrogen, more than 19 free amino acids including several essential amino acids, and more than 16 free mononucleotides. The p-subvessel in the node contains basophile granules, small p-microcell, and somewhat large, round p-microcells. Cells with pale cytoplasm are also found, and the cytoplasm often contains many chromaffin granules. Kim also reported the presence of copper, magnesium, calcium, iron, manganese, zinc, and cobalt [7].

## 6. Primo-Vascular Systems

Kim recognized five different primo-vascular systems comprised primo-nodes and vessels as follows. (1) Internal PVS contains internal primo-vessel and internal primo-nodes, which is distributed in all the blood vessels, lymphatic vessels, and the cardiac cavity. The internal primo-vessel is very fragile, and its external jacket and connective tissue are weakly developed. The internal primo-node has a structure particularly similar to that of the hematopoietic organ, and myeloid and lymphoid cells are present in the network of the reticular tissues. Cells similar to those found in parenchymal organs are also occasionally identified. (2) The intraexternal PVS includes intraexternal primo-vessel and intraexternal primo-node and extends separately of the neighboring organs, the blood, lymphatic vessels, and nerves. In the intraexternal primo-vessel, the connective tissue and the external jacket are, usually, more developed as compared to the internal primo-vessel. The primo-vessel sinus of the intraexternal primo-node includes cells with a light cytoplasm and also basophile structures. (3) The external PVS contains external primo-vessels and external primo-nodes and primarily passes around vessels and nerves. A thick connective tissue membrane covers the external primo-vessel. The primo-vessel sinus of the external primo-node includes a large number of chromaffin granules. (4) The neural PVS comprises neural primo-vessels and neural primo-node, and it is immersed in the cerebrospinal fluid of the central nervous system. Its branches are dispersed not only in the parenchyma of the central nervous system but also in the peripheral nerves. (5) The intraorgan PVS is located inside the organs. They include intraorgan primo-nodes, terminal primo-nodes, and terminal p-subvessels. They are the intraorgan elements of the internal primo-nodes, external primo-vessels, and neural primo-vessels. Many primo-vessels are joined in the intraorgan primo-node to the terminal p-subvessels, which are connected directly with cell nuclei.

Different primo-vascular systems are connected with one another. The internal PVS connects with the intraexternal PVS through the blood vessel wall and with the external PVS via the external primo-node. The intraexternal PVS communicates with the external PVS through the external primo-node and it is connected with the neural PVS. All the systems are totally interrelated to each other [7].

### 6.1. Histochemistry of PVS

In order to characterize the cellular and molecular composition of PVS, Kim used various staining techniques, which were largely practiced at his time as immunohistochemistry is practiced today. Kim did not describe the stains that he used in his research. Rather he only mentioned the stain names, because their use and properties were well known and did not require special explanations. Most of the stains that Kim employed in his experiments are still in use today and are commercially available. For example, Kim used Feulgen stain to visualize nucleic acids in PVS [6, 7]. The Feulgen staining remains the standard for precise imaging of DNA [37].

### 6.2. Nucleic Acids

**Figure 3:**
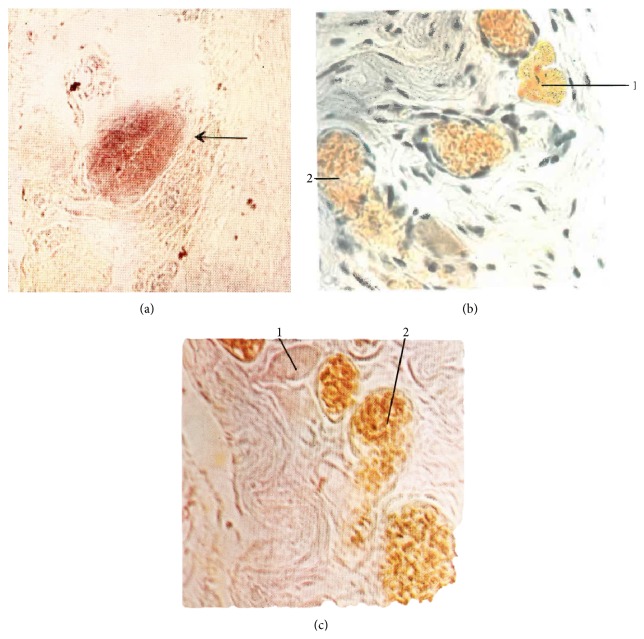
Superficial primo-node. (a) Feulgen stain. Sinus of the superficial primo-node (arrow) (×160). (b) Hillarp-Hokfelt stain. 1: chromaffin cell, 2: blood vessel. (c) Sevki stain; 1: chromaffin cell; 2: blood vessel [7].

Kim used Feulgen, Unna-Pappenheim, Brachet, acridine orange, and hematoxylin-eosin stains to characterize nucleic acid distribution in PVS (Table 1). The Feulgen stain was used to visualize cell nuclei, basophile granules, and other structures containing DNA inside p-subvessels by virtue of its capacity to penetrate the cellular membrane. He also imaged basophile particles and p-microcells in the sinuses of primo-nodes (Figure 3(a)). As a result of acid hydrolysis of DNAs [38], it stains them red as showed in many images of endothelial cell nuclei in walls of p-subvessels. The elongated, rod-like nuclei of p-subvessel endothelial cells work as a unique marker of PVS. In Kim's studies, the DNA of p-microcell nucleosome was stained by Feulgen reaction while DNA in p-microcell nucleoplasm was revealed by the Brachet stain [7].

Kim used acridine orange in his earlier experiments to visualize nucleic acids in nonfixed PVS samples. “*Corpuscles and ducts were extracted and stained with acridine orange, and examined under a luminescent microscope. The inner substance of the corpuscle and the duct linked to it selectively fluoresced brilliantly: in blue green or yellowish green. This fact has convinced us that an enormous amount of DNA is contained in the inner substance of the corpuscle and the duct*” [6]. Acridine orange is used as a nucleic acid-selective fluorescent cationic dye. Being cell-permeable, it binds with DNA and RNA [51]. A light with a maximum wavelength of 502 nm excites the acridine orange/DNA complex which emits at the maximum wavelength of 525 nm (green). When it interacts with RNA, the excitation shifts to 460 nm and the emission shifts to 650 nm (red) [57]. However, acridine orange also is accumulated in mast secretory granules and other cellular acidic compartments and emits red light [53, 54].

When Kim studied regeneration of injured tissues, he observed newly formed round p-microcells that contain clear chromatin particles. These basophile structures were stained deep violet by hematoxylin and show a strong positive Feulgen reaction [7].

### 6.3. Chromaffin Cells, Epinephrine, and Norepinephrine

Kim identified chromaffin cells in the internal substance of superficial primo-nodes, primarily in its superior and central parts [7]. Chromaffin cells congregate in small groups close to the sinus around the blood vessels. These cells vary in shape and size, and the location of their nuclei is not well defined. In some instances, chromaffin granules are collected together both within and outside the sinus. These granules are positive in the Hillarp-Hokfelt and Sevki stains (Table 1). They are dispersed around the blood capillaries in the internal substance of the node (Figures 3(b) and 3(c)).

### 6.4. Fibers

**Figure 4:**
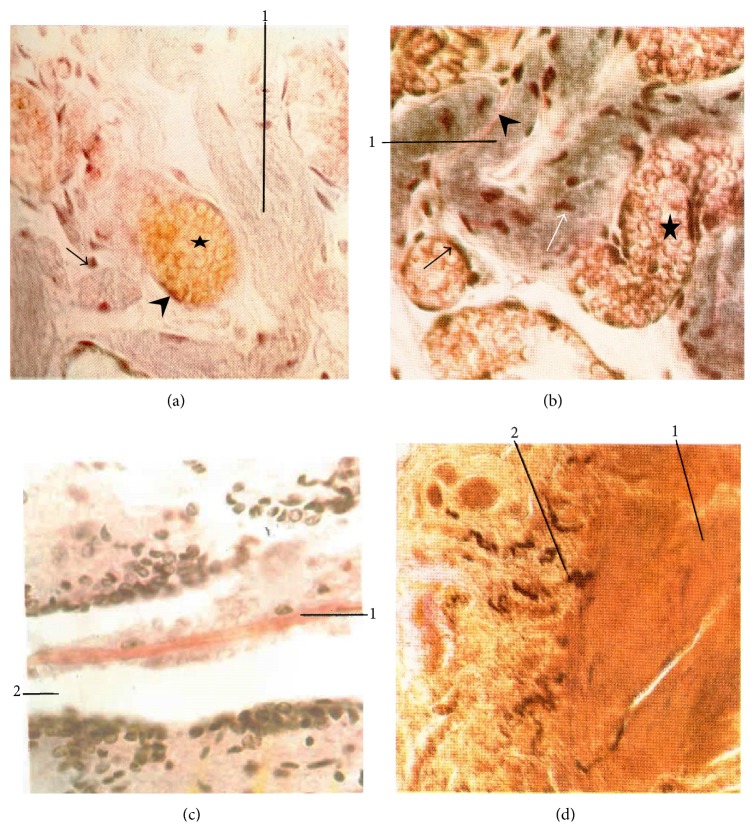
Fibers. (a) Superficial primo-node (resorcin-fuchsin stain) (×400). 1: sinus, arrow: elastic fiber, arrowhead: elastic membrane in blood vessel, and star: erythrocytes. (b) Superficial primo-node (Verhoeff stain) (×400). 1: sinus, white arrow: basophil particle, star: erythrocytes, blood vessel membrane: black arrow, and collagen fiber between sinus folds: arrowhead. (c) Neural Bonghan duct (in the central canal of the spinal cord) (Van Gieson stain) (×400). 1: primo-vessel, 2: central canal of the spinal cord. (d) Nerve-supply at the superficial primo-node (Gros-Schultze reaction) (×160). 1: superficial primo-node, 2: nerve fiber [7].

Large amount of fibrous connective tissue was found between sinuses in primo-nodes. The primo-nodes are partly filled with collagen fibers that are produced by reticular cells. Reticular fibers intersect to create a thin net that serves as a supporting mesh in the primo-nodes and vessels. Kim found that this connective tissue contains collagenous, elastic, and argyrophilic fibers. To characterize these materials histologically, Kim used resorcin-fuchsin, Van Gieson, Verhoeff, and Gros-Schultze stains (Table 1) [7]. A large number of fibers were found on the external envelope of the sinus, which were stained by the resorcin-fuchsin (Figure 4(a)). The jacket is folded in a distinctive way and appears light purple in color. The elastic fibers that are perpendicular to the slide and along the outer surface of the sinus together with the outer membrane of blood vessels appear dark purple. Erythrocytes counterstained with eosin appear yellow-red. The details of the sinus in primo-vessels are more pronounced with Verhoeff stain (Figure 4(b)). The elastic fibers of the sinus appear transparent black. The sinus is folded, and the collagen fibers between the folds are stained red. Basophil particles cover the sinus surface. The blood vessels filled with erythrocytes surround the sinus, and a dark purple color distinguishes the outer membranes of blood vessels.

Kim used Van Gieson and Gros-Schultze stains to visualize the neuronal primo-vessels. He described the live neuronal primo-vessels as semitransparent, milk-white, and of a very delicate texture [7].  Figure 4(c) shows the neural primo-vessel in the central canal of the spinal cord stained by Van Gieson stain, showing red collagen fiber. The nerve fibers were imaged in the superficial primo-node by a Gros-Schultze stain (Figure 4(d)).

### 6.5. Hematopoietic Stem Cells

**Figure 5:**
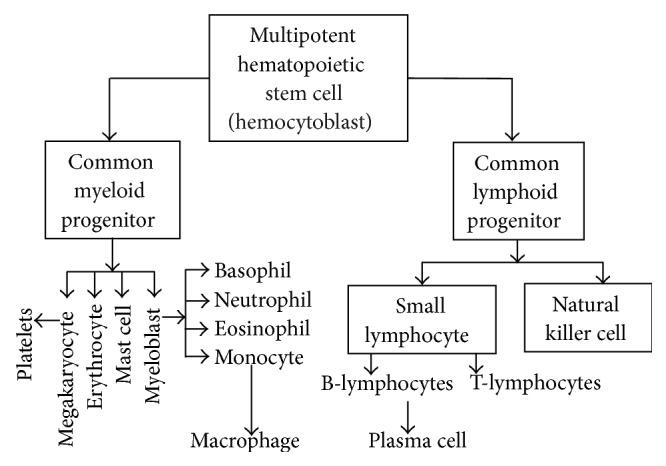
Hemocytoblast cells generate two major progenitor cell lineages, myeloid and lymphoid progenitors.

All cellular blood components are formed from hematopoietic stem cells. In developing embryos, blood formation occurs in aggregates of blood cells in the yolk sac, called blood islands [58]. Maximow was the first who coined the word “stem cell” for the initial cell that gives rise to all blood cells. He used the cell name hemocytoblast for the multipotent hematopoietic stem cell. He also discovered that hemocytoblast generates two independent series of blood cells: myeloid and lymphoid systems. Furthermore, Maximow determined that as the development progresses, blood formation occurs in the spleen, liver and lymph nodes, and later in the bone marrow [26, 27]. The common myeloid progenitor produced from the hemocytoblast differentiates into four cell classes: erythrocytes, mast cells, megakaryocytes (which form platelets), and myeloblasts. Myeloblasts differentiate into basophils, neutrophils, eosinophils, and monocytes, which later mature into macrophages. Lymphoid progenitors differentiate into natural killer cells and lymphocytes (B cells and T cells). B cells differentiate into plasma cells [59] (Figure 5).

**Figure 6:**
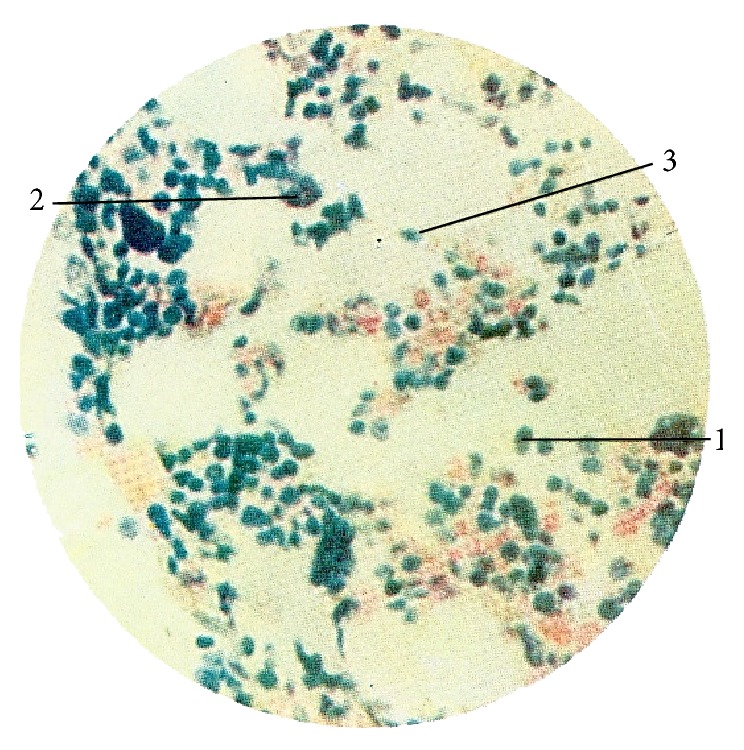
Internal primo-node (Giemsa stain). 1: hemocytoblast, 2: megakaryocyte, and 3: reticular fiber [7].

By using a variety of stains and biochemical tests, Kim discovered that internal primo-nodes contain cells that are typical for the hematopoietic organs. “*These are myelopoietic and lymphogenetic cells in different stages of differentiation, that is, granulopoietic, monopoietic, erythrogenic and lymphopoietic elements and megakaryocytes*” [5, 7]. These are the same two major progenitor cell lineages and cell classes shown in Figure 5. Kim gave an example by revealing the cellular composition of the internal primo-node by using the Giemsa stain. Giemsa stain has a remarkable dynamic range of colors capable of visualizing the complex morphological composition of blood cells (Table 1) [43, 60–62].  Figure 6 identifies a multipotent hematopoietic stem cell, hemocytoblast, and an important member of myeloid lineage, megakaryocyte [5, 7]. He described other cells differentiated from both myeloid and lymphoid progenitors. “*This suggests that an active hematopoietic process takes place in the internal Bonghan corpuscles*” [7]. Kim designed and carried out an elegant experiment to prove this last statement. In his experimental design, Kim used the known effects of phenylhydrazine on the hematopoiesis. When the erythrocytes in the bone marrow and peripheral blood are destroyed with phenylhydrazine, erythropoiesis is increased [63, 64]. Kim also challenged rabbits with the phenylhydrazine and observed a striking increase in production of erythrocytes. He explained this effect by activated internal primo-nodes, which were significantly enlarged after the phenylhydrazine treatment. Quite the opposite, anemia happened progressively when the internal PVS was injured. These data provide the evidence that the hematopoiesis is one of the essential functions of the internal primo-vascular system.

It is important to note that the effects of phenylhydrazine on the hematopoiesis have been reported recently in rats and mice [65, 66]. Both research groups observed phenylhydrazine-induced erythropoiesis. However, these researchers could not reconcile the large damage on bone marrow and the increased production of erythrocytes. This phenomenon could be easily explained by the amplified hematopoietic activity arising in the PVS.

### 6.6. Multipotent Stem Cells

James Till and Ernest McCulloch, while studying the effect of radiation on the bone marrow of mice at the Ontario Cancer Institute, Toronto, demonstrated the presence of self-renewing cells in mouse bone marrow [67, 68]. At the same time, 6500 miles from Toronto, Bong Han Kim reported that he had isolated p-microcells from the newly discovered primo-vascular system and induced their proliferation under artificial conditions. He stated that mature p-microcells had a cell-like structure, contained chromosomes, and participated in the tissue regeneration [7]. Each of these properties belongs to stem cells [59, 69]. Kim also demonstrated that p-microcells were harbored in a primo-node, a highly vascular organ that provides physiological conditions favorable for p-microcells [7], as well as a stem cell niche [70]. The role of a primo-node as a stem cells niche was discussed again by PVS researchers [71–73].

#### 6.6.1. Morphology

**Figure 7:**
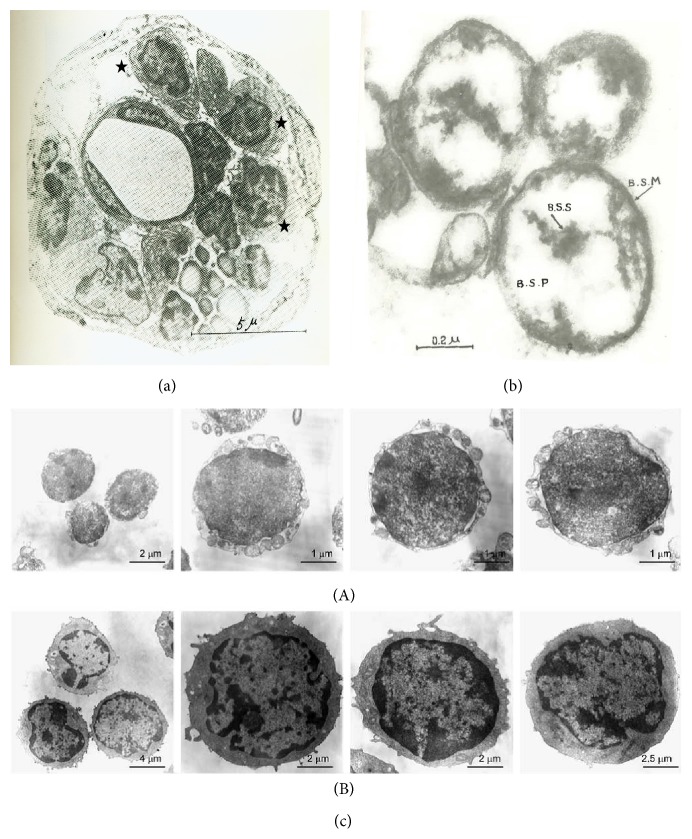
TEM micrographs of p-microcells, very small embryonic-like (VSEL), and hematopoietic stem cells. (a) External primo-node. Star: p-microcells. (b) P-microcells. BSS: p-microcell nucleosome, BSP: p-microcell nucleoplasm, and BSM: p-microcell membrane [7]. (c) TEM of very small embryonic-like (VSEL) cells and hematopoietic stem cells. (A) Small embryonic-like (VSEL) cells are small and measure 2–4 *μ*m in diameter. They possess a relatively large nucleus surrounded by a narrow rim of cytoplasm. The narrow rim of cytoplasm possesses a few mitochondria, scattered ribosomes, small profiles of endoplasmic reticulum, and a few vesicles. The nucleus is contained within a nuclear envelope with nuclear pores. Chromatin is loosely packed and consists of euchromatin. (B) In contrast hematopoietic stem cells display heterogeneous morphology and are larger. They measure on average 8–10 *μ*m in diameter and possess scattered chromatin and prominent nucleoli (reprinted by permission from Macmillan Publishers Ltd. leukemia [36], copyright, 2006).

Kim studied the structure of p-microcell, the electron microscope.  Figure 7(a) shows p-microcells inside a small external primo-node. Kim thoroughly described p-microcells found within the node. The p-microcells vary in shape. Some are round and others irregular, and their membranes are smooth. In the cytoplasm of some of the round cells, endoplasmic reticulum is well developed; it is organized around the nucleus and mitochondria. In the cytoplasm of the cells, one nucleus or two nuclei can be found in the middle or periphery of the cell. These nuclei have thin membranes and the abundant chromatin, and the nucleoli are located down in the middle of the nuclei. The nuclear membrane occasionally is wrinkled. The boundary of the cell is smooth or occasionally has processes. In the cytoplasm, there are numerous vacuolar structures. The cytoplasm of some cells includes vesicular endoplasmic reticulum and many small round granules of high electron density. However, such granules cannot be noticed in the vacuoles. Mitochondria of an oblong shape are normally found in a part of the cytoplasm. Adjoining cells come close to each other or are linked to one another via small cytoplasmic processes [7].

Isolated p-microcells at a higher magnification are shown in Figure 7(b). Kim found that the p-microcell was normally round but often oval in shape. A normal p-microcell was 1.2–1.5 microns in size, while the smallest one was 0.8 micron and the largest one, which was not often observed, was 2.4 microns. P-microcell had a thin membrane of high density with very distinct contour. The nucleus took various forms. The p-microcell membrane was very dense and had a distinctive silhouette. Granules of various sizes and relatively high electron density were seen in the cytoplasm next to the p-microcell membrane.  Figure 7(c) shows a TEM of very small embryonic-like (VSEL) cells and hematopoietic stem cells obtained by Kucia et al. from murine bone marrow by multiparameter sorting [36]. We noticed (Figure 7(c)) that very small embryonic-like (VSEL) cells as compared to hematopoietic stem cells were smaller in size (2–4 versus 8–10 *μ*m). They contain relatively large nuclei and a narrow rim of cytoplasm. The authors hypothesized that this population of very small embryonic-like (VSEL) stem cells was deposited early during development in bone marrow and could be a source of pluripotent stem cells for tissue/organ regeneration. It should also be noticed that p-microcells are similar by morphology to these cells [36].

#### 6.6.2. Composition

The biochemical properties of p-microcell were carefully analyzed by Kim. The p-microcells were found to contain 2.5 × 10^−13^ g of DNA, 1.2 × 10^−13^ g of RNA, and 1.7 × 10^−12^ g of protein. The molecular weight of DNA in p-microcell was estimated at 1.8 × 10^6^–3.0 × 10^6^ KD. The most of DNA (99.8%) is in p-microcells. Kim suggested that p-microcells mature into cells within primo-vascular system [7].

#### 6.6.3. Cell Culture, Cell Formation from p-Microcells

Kim developed a culture medium that mimics p-fluid by composition, physical, and biochemical properties. He was able to demonstrate the cultivation, proliferation, and fusion of p-microcells leading to the formation of cells in artificial conditions. The entire process was recorded by the time-lapsed photography. The process of the cell formation was described as being composed of the stage of proliferation and the stage of fusion. The p-microcells proliferate by the formation of numerous daughter cells arranged as clusters of cells. Then the p-microcells in the same cluster of cells fuse so that the nuclei of combined p-microcells coalesce into the nucleus of one relatively large cell. Meanwhile, their cytoplasm merges to produce the cytoplasm of a newly created large, mature p-microcell. We speculate that a large mature p-microcell is equivalent to multipotent stem cell. At given conditions, a large, mature p-microcell can be divided back into p-microcells. The number of p-microcells at the time of cell division is similar to the number of chromosomes [3, 7].

#### 6.6.4. *In Vitro* Proliferation of p-Microcells

In order to observe and quantify* in vitro* proliferation of p-microcells, Kim obtained them from the internal primo-node of a rabbit and were cultivated for various lengths of time. The culture was studied under a phase contrast microscope, and the biochemical analyses of cells were also performed. It had been detected with the phase contrast microscope that initially nearly all the p-microcells were of 1.0–1.5 microns in size. Numerous daughter p-microcells were created from the mother p-microcell within 48 hours. After 72 hours, the multiplied p-microcells were fused into many clusters and, after 144 hours, a large number of cells were in the formation of cytoplasm stage. For the duration of the formation of mature p-microcells from daughter p-microcells, the content of DNA, RNA, and protein increased by factors 16, 9, and 32, respectively. The content of DNA increased rapidly from the start to 72 hours and later the increase was much slower. These data were compared with the morphological transformations detected by the phase contrast microscope. The biosynthesis of DNA occurred primarily within the phase of proliferation of mother p-microcell into daughter p-microcell and the synthesis of DNA barely takes place while p-microcells are fused to form a nucleus and then cytoplasm of mature p-microcells. Contents of protein and RNA, on the other hand, display a gradual increase after 72 hours of cultivation. These results correlate with the morphological findings on the formation of cytoplasm and growth of the mature p-microcell [3, 7]. Accepting the fact that one p-microcell carries one chromosome (see Section 7), we can estimate the frequency of proliferation and formation of new cells from p-microcells. The rabbit has 44 chromosomes and 88 DNA molecules in one adult cell [74]. Because DNA content increased 16 times during 6 days of p-microcell culture [7], the frequency of proliferation and forming of new cells from p-microcells is equal to 16/(88 × 6) = 0.03 or 3%. This value agrees well with the frequency of proliferation of the mice CNS progenitor cells into the multipotent CNS neurospheres that are found to be in a range of 1.5–3.3% [75]. The frequency of proliferation and cell forming found by Kim [7] is in a good agreement with the recently reported frequency of forming neurospheres from the p-microcells obtained from the mouse's intravascular primo-nodes and vessels [76]. The authors stated that they produced 176 spheres from 1000 cells per 7 days. These results make the sphere forming the frequency equal to 176/(1000 × 7) = 0.025 or 2.5%.

#### 6.6.5. Distribution and Circulation of p-Microcells

P-microcells at different levels of maturity were identified only in the primo-vascular system: in the primo-vessels and primo-nodes. In order to examine distribution and circulation of p-microcells, P32-tagged microcells were utilized and traced by microautoradiography. P-microcells were removed from the superficial nodes, internal primo-vessels, and tissue cells. Tagged p-microcells were injected into the internal primo-vessels of organs and radioautography was used to trace the circulation course of primo-fluid and to the various tissues of internal organs [3, 7].

30 minutes following the injection, a few labelled mature p-microcells of 3-4 microns in size, spherical or oblong, were detected individually or grouped among the lung alveolar epithelial cells. Typically, p-microcells did not mature for up to 12 hours after the injection. However, they were proliferated and grown. In the alveolar epithelial cells, mature p-microcells and basophile, measuring 5-6 microns and which had no visible cytoplasm, were observed labelled in 24 hours following the injection of tagged p-microcells. 48 hours after the injection, alveolar epithelial cells of an almost matured state were found. They were just like the neighboring alveolar epithelial cells; their cytoplasm was quite revealing, and their nuclei were strongly stained with basic dyes. At this time, the tagged p-microcells at various phases of development could be identified [3, 7].

Similar results were obtained with injection of labeled p-microcells into other internal organs: liver, kidney, and ovary. 48 hours after injection, the labeled organ cells could be seen among the endogenous organ cells. These experimental results were completely validated by control experiments. When the suspension incorporating the same quantity of labelled p-microcells as in the PVS tests was injected into blood vessels, the obtained results were very different [3, 7].

In summarizing the labeled p-microcell experiments, Kim made the following conclusions: (1) P-microcells created in the internal organs move into the superficial primo-nodes and after some time return once again to the internal organs. The amount of DNA found in one young p-microcell is nearly equal to that contained in one chromosome. When an adult cell generates p-microcells, the number of p-microcells is equal to the number of chromosomes. (2) P-microcell injected into the superficial primo-node becomes larger and becomes a mature p-microcell on the way to various internal organs via primo-vessels. Then they move through intraorgan primo-vessels and grow into cells within the internal organs. Moreover, p-microcells injected into the superficial primo-node move to the external, intraexternal, and internal primo-nodes. (3) P-microcell of the superficial primo-node, when injected into the primo-vessel, grows into cells in the internal organs. These results mean that p-microcells produced in the organ cells are transported to the superficial primo-nodes through primo-vessels and after that onto the profound primo-nodes and that they form organ cells, passing through different intraorgan nodes [3, 7].

#### 6.6.6. Potency of p-Microcells

It was clear to Kim that mature p-microcells are stem cells and the primo-nodes and vessels collect, harbor, mature, and distribute these cells. He exemplified this point in the description of the hematopoiesis in the internal primo-nodes, where the hemocytoblast, multipotent stem cell, gives rise to myeloid and lymphoid progenitors (see Section 6.5). Kim demonstrated that mixture of p-microcells taken from the superficial nodes, internal primo-vessels, and tissue cells could differentiate into tissue cells of various organs (see Section 6.6.5). However, what is the potency of individual p-microcells removed and cultured from various superficial primo-nodes? In answering this question, Kim was guided by the old and well-established knowledge in acupuncture showing that superficial primo-nodes (acupoints) in different locations possess particular associations with relevant internal organs. Therefore, different cells would be created from p-microcell extracted from different nodes. Kim cultivated 344 samples of the p-microcells taken from the superficial primo-nodes in 79 areas of the human body. As was anticipated, different cells were produced according to the location of those superficial primo-nodes. It means that the p-microcells created in the tissue cells of various organs move through the superficial primo-nodes linked with the internal organs. In PVS, the primo-fluid has many circulating channels, and each channel is linked with a different organ; however some organs may have connections with many circulating channels while some with fewer channels. It means that mature p-microcells taken from adult superficial primo-nodes can differentiate into a few different organ cells [3, 4, 7]. In this sense, they are playing a role of multipotent stem cells.

#### 6.6.7. Regeneration of Injured Tissues

Regeneration of injured tissue was demonstrated with a normal liver of a rabbit that was injured with a glass 2 mm capillary tube, and histological samples were prepared at different time intervals. The regeneration of the injured liver was described to occur in a few phases as follows. (1) At 12 hours following the injury, inflammatory processes (i.e., hemorrhage, accumulation of leukocytes) occurred. The injured liver tissue progressively experienced changes in its stainability and became necrotic at the point of injury. (2) 24 hours after the injury, hemorrhage and buildup of leukocytes began to disappear slowly and karyolysis occurred in the necrosed cells with the injured cells and then disappear entirely. (3) Next, a cluster of mature p-microcells of 3–5 microns in size are detected surrounding the area of the injury. (4) The mature p-microcells assume round-like shapes. (5) Profoundly stainable cytoplasm took shape around the spherical nucleus of the mature p-microcells and (6) the cells developed further and grow to a size resembling normal liver cells [3, 7].

## 7. Chromosome Recycling

Dr. Kim described cell death as a part of the continuous “cell-*p-microcell* and* p-microcell*-cell” cycle. The steps in the cycle include the following: chromatin condensation and packaging into membrane-bound granules; deformation and rupture of nuclear membrane and granule release into the cytoplasm; and external membrane rupture that releases the granules to the outside. These granules preserve the parent cells' DNA and are subsequently used for generation of mature p-microcells (stem cells) [7]. That process, described by Kim, is almost identical to apoptotic cell death and the generation of apoptotic bodies with a major difference: apoptotic bodies are destroyed by phagocytic cells [77]. Important to note is that it was recently demonstrated that, under specific conditions, DNA can be transferred from one cell to another by phagocytosis of apoptotic bodies [78–80].

Kim's experiments on the adult cell conversion into a stem cell provoke a question of whether differentiated somatic cells can generate stem cells. Recent studies describe the reprogramming of terminally differentiated somatic cells to pluripotent stem cells by transducing adult cells with a limited set of defined transcription factors [81]. What is important and is mentioned a few times in Kim's publication is that the amount of DNA contained in one p-microcell is nearly equal to that contained in one chromosome [3, 7]. It is easy to verify that this statement is true by using experimental data presented in [7]. P-microcells were collected from primo-vessels and nodes of rabbit with a thin glass capillary driven by a micromanipulator. The content of DNA in one p-microcell was found to be (2.6 ± 0.6) × 10^−13^ g. The molecular weight of DNA in p-microcells was (2.4 ± 0.6) × 10^9^ D. Consequently, the concentration of DNA in one p-microcell is 2.6 × 10^−13^/2.4 × 10^9^ ≈ 1.1 × 10^−22^ moles. Therefore, the number of DNA molecules in one p-microcell is 1.1 × 10^−22^ × 6 × 10^23^ = 66 ± 32 molecules. The number of chromosomes in rabbit is 44 [74]. The number of DNA molecules per chromosomes is equal to 88. Assuming that Kim used about 10 (or less) samples for his measurements, one can say with 95% confidence that one p-microcell contains one chromosome. It is interesting to note that microcells, cytoplasmic fragments that contain micronuclei composed of one or a few chromosomes, can be generated directly from adult cells [82].

It will take considerable efforts to repeat and validate the “cell-*p-microcell* and* p-microcell*-cell” cycle that has been proven by Kim with very high confidence using a radioactive labeling [3, 7].

## 8. Future of the Primo-Vascular System

We strongly believe that complete characterization of the PVS will fully confirm the existence of this vast, distinct vascular system, which will soon create a new paradigm in biology and medicine. It will bring together western and eastern medical philosophies, provide an unlimited source of multipotent stem cells, and bring new diagnostic and therapeutic methods. The highest potential impact is expected in acupuncture [83, 84], osteopathic manipulative medicine [85, 86], pain management, developmental biology [87], tissue regeneration, organ reconstruction, diabetes, and cancer prevention and treatment [88].

## 9. Comment on Terminology

In Section 3 we showed that different parts of the primo-vascular system and the whole system have been described by different scientists under different names. It is very essential that all PVS researchers use the same terminology. It was an important step forward when a new unified terminology for primo-vascular system was proposed [10]. This nomenclature does not include any personal name and allows it to be used internationally without any reservations. This new terminology does not diminish the contribution of Bong Han Kim because his role is acknowledged practically in all PVS publications. Therefore, trying to introduce another new terminology to the same system and its components would only incur the confusion without giving any benefits to this new emerging field.

## 10. Conclusions

1. According to Kim, an extensive vascular system, named as primo-vascular system, different from blood and lymphatic vascular system, exists in animals and human. The vascular cell system is distributed throughout the entire body, over and inside of various organs, inside and outside of the blood and lymphatic vessels, in the internal and the peripheral nervous system, and in the corium or in the subcutaneous layers of the skin. It is comprised of two structural elements: vessels and nodes. The vessel is a bundle of 1–20 subvessels of 3–25 *μ*m in diameter laid into an external jacket.
2. The primo-nodes have various shapes elements (round, oval, or multifaceted). They are of 0.1–1.6 mm in size. A vessel bundle of the incoming (afferent) vessels enter into the node, branch into additional bundles, and fill the node interior by tightly spun and folded bundles. Subvessels converge and exit from the node as efferent primo-vessels. The enlarged p-subvessels inside the node, which are called the sinuses of the node, harbor microcells, the progenitors of multipotent stem cells.
3. A series of primo-nodes and vessels form a circulatory system that is composed of many circulatory channels. Each channel is linked with a different organ; however some organs may have connections with many circulating channels while some with fewer channels. The individual channel may begin with the superficial node; then, after being successively connected with a few profound nodes, it links with the intraorgan terminal node and then closes by the channel that comes back to the superficial node.
4. A special fluid moves through the circulating channels and carries progenitors of multipotent stem cells (microcells), hormones, amino acids, lipids, sugars, proteins, and hyaluronic acid. Electrical signals are also shown to travel along these channels. The progenitor stem cells turn into multipotent stem cells within sinuses of the nodes. Being delivered to the internal organ, they differentiate into new organ-specific cells. In turn, the aged (or injured) organ cells are converted into microcells.
5. Stimulating superficial nodes by acupuncture or osteopathic manipulative techniques results in sending electrical signals, hormones, and multipotent stem cells to a connected organ. These mediators support the organ stimulation and regeneration.
6. The future characterization of the primo-vascular system will bring better understanding of the mechanisms and underlying techniques of acupuncture and osteopathic manipulative medicine. This knowledge will benefit pain management, developmental biology, tissue regeneration, organ reconstruction, diabetes, and cancer prevention and treatment.

## Figures and Tables

**Table 1:** Stains used by Bong Han Kim.

Stain	Target	Color	Mechanism	Reference
Feulgen	Cell nuclei, basophile granules, and other structures containing DNA inside p-subvessels. Basophile particles and p-microcells in sinuses of primo-node. Endothelial cell nuclei in walls of p-subvessels. DNA of p-microcell nucleosome.	DNA is stained red 570 nm. The background, if counterstained, is green.	Acid hydrolysis of DNA.	[6, 7, 37, 38]

Hillarp-Hokfelt	Chromaffin cells, epinephrine (adrenaline), and norepinephrine (noradrenaline). Small granules inside p-subvessels.	Yellow.	Oxidation of adrenaline and noradrenaline with potassium iodate gives a pigment formation. The method is based on the formation of iodochromes of the hormones.	[7, 39]

Sevki	Chromaffin cells, epinephrine, and norepinephrine.	Chromaffin cells: bluish-red; inside primo-node: red to bluish-red; red blood cells: brown; mast cell granules: red.	Sevki stain (~6% water dilution of Giemsa stain).	[7, 40–42]

Giemsa	Adrenal medullary cells, chromaffin cells, epinephrine (adrenaline), norepinephrine (noradrenaline), collagen, erythrocytes, platelets, lymphocytes, monocytes, megakaryocytes, and hemocytoblasts.	Chromaffin: brown; collagen: blue; erythrocytes: pink; platelets: light pale pink; lymphocyte: sky blue; monocyte: pale blue; leukocyte nuclear chromatin: magenta; megakaryocytes: reddish-blue nuclei and blue cytoplasm; hemocytoblasts: large, vesicular, pink nuclei, and prominent nucleoli. Giemsa, after a dichromate fixation, produced a green color.	Giemsa's solution is a mixture of methylene blue, eosin, and Azure B. Methylene blue is a cationic dye. It binds to tissue anions and stains basophilic substances, including nucleic acids. Eosin is an anionic dye and is attracted to positively charged protein groups (cations), such as amino groups. It is an acidophilic stain. Azure B is formed by the oxidation of methylene blue and is a basic stain.	[7, 40, 43–45]

Gros-Schultze	Nerve fibers and nerve endings.	Dark brown to black.	Silver nitrate bath. The silver nitrate is reduced by the sodium potassium tartrate into a metal silver that is adsorbed by argyrophilic nerve fibers.	[7, 46, 47]

Van Gieson	Neural vessels. Collagen.	Collagen: pink or deep red; cytoplasm: yellow; elastic fibers: blue to black.	Mixture of picric acid and acid fuchsine (picrofuchsin). The method is based on the affinity towards elastic fibers displayed by the dye resulting from a reaction between resorcin and basic fuchsine in the presence of ferric chloride.	[7, 43, 48]

Verhoeff	Elastic fibers, nuclei, and collagen.	Elastic fibers: intense blue-black to black; nuclei: blue to black; collagen: red; other: yellow. Eosin counterstain shows erythrocytes red.	^a^Combination of stains: hematoxylin, iron(III) chloride, Lugol's iodine, Van Gieson's stain (acid fuchsine, picric acid), and sodium thiosulfate. The tissue is stained with a hematoxylin, ferric chloride, and iodine.	[7, 49, 50]

Unna-Pappenheim	RNA and DNA in tissue sections.	RNA: red; DNA: green.	^ab^Methyl green-pyronin combination. Competition between the slow staining, but doubly charged, methyl green and the more rapidly staining, singly charged pyronin Y.	[6, 51, 52]

Brachet	DNA, RNA, and DNA in the nucleus of cells and RNA in the nucleolus. RNA in the cytoplasm of cells. DNA in p-microcell nucleoplasm.	DNA: green; RNA; p-microcell nucleoplasm: Red.	Methyl green-pyronin combination. Similar to that of Unna-Pappenheim method. Brachet introduced control by using RNase solution before staining.	[7, 50, 51]

Acridine-orange	Vital stain for DNA and RNA. Primo-vascular nodes and vessels. Acridine orange also accumulates and emits red light in mast secretory granules and other cellular acidic compartments.	RNA: fluorescent red; DNA: fluorescent green; mast cells: red.	Acridine orange is a basic dye. Basic dyes are cationic and will stain anionic or acidic molecules. Staining is pH sensitive. Acidic substances that stain with basic dyes are termed basophilic.	[6, 51, 53, 54]

Hematoxylin-eosin	The basophilic structures containing nucleic acids, such as the ribosomes and the chromatin-rich cell nucleus, and the cytoplasmic regions rich in RNA. The eosinophilic structures composed of intracellular or extracellular protein. Most of the cytoplasm is eosinophilic.	Hematoxylin colors basophilic structures with blue-purple hue and alcohol-based acidic eosin colors eosinophilic structures as bright pink. Red blood cells, collagen fiber: red.	Oxidized hematoxylin (hematein) has a selective affinity for nuclei when combined with aluminum ion. The mechanism of eosin staining is not fully understood but is believed to be of an electrostatic nature. Negatively charged eosin ions will stain positively charged tissue ions.	[7, 43, 50, 55]

Resorcin*-*fuchsin	Glycogen, basement membrane, reticulum fibers, collagen, and other structures containing polysaccharides. flexible fibers, and collagen inside primo-nodes.	Elastic fiber: purple; elastic membrane in blood vessel: dark purple; nuclei: pale red; red blood cells: red if counterstained by eosin.	Acetylation, sulfaction, and phosphorylation induce binding of resorcin*-*fuchsin.	[7, 43, 56]

^a^ *Verhoeff mechanism*: the differentiating is accomplished by using excess of ferric chloride to break the tissue-ferric chloride dye complex. The dye will be attracted to the larger amount of ferric chloride in the differentiating solution and will be removed from the tissue. The elastic fibers have the strongest affinity of the iron-hematoxylin complex and will retain the dye longer than the other tissue components. Van Gieson's solution is used as a counterstain. ^b^ *Unna-Pappenheim mechanism*: methyl green has two cationic charged groups that become linked to the phosphate moieties in the DNA. The pyronin Y displaces the methyl green from all sites of linkage except where its double charge gives it a selective advantage (acidic polymer such as DNA). Consequently the methyl green stains DNA and retains its binding to this substance against the competitive action of pyronin Y. Pyronin Y stains the less polymerized RNA rapidly, and it can displace methyl green from linkages having smaller polymeric acidic substances (RNA).
